# Near-complete inhibition of rumen methanogenesis via microbial and enzymatic modulation using a low dose of *Asparagopsis taxiformis* combined with 3-nitrooxypropanol

**DOI:** 10.1186/s40104-026-01430-x

**Published:** 2026-06-05

**Authors:** Shuai Li, Yi Sun, Xiong Tong, Zhifei Zhang, Xinyan Ma, Dagang Li, Li Min

**Affiliations:** 1 Ministry of Agriculture Key Laboratory of Animal Nutrition and Feed Science in South China, Guangdong Public Laboratory of Animal Breeding and Nutrition, Institute of Animal Science, Guangdong Academy of Agricultural Sciences, Guangzhou, China; 2 State Key Laboratory of Animal Nutrition, Institute of Animal Science, Chinese Academy of Agricultural Sciences, Beijing, China; 3 Southern Marine Science and Engineering Guangdong Laboratory (Zhuhai), Zhuhai, China; 4 Agri-Food and Biosciences Institute, Hillsborough, UK

**Keywords:** *Asparagopsis taxiformis*, Hydrogenotrophic methanogenesis, Methane inhibition, Metagenomics, 3-Nitrooxypropanol, Rumen fermentation

## Abstract

**Background:**

Enteric methane (CH_4_) from ruminants represents a major contributor to agricultural greenhouse gas emissions. The red seaweed *Asparagopsis taxiformis* (*A. taxiformis*) is a highly effective CH_4_ emission inhibitor, but its large-scale application is restricted by limited biomass availability. This study evaluated whether reducing the inclusion level of *A. taxiformis* (0.32% dry matter, DM) combined with 3-nitrooxypropanol (3-NOP; 0.05% DM) could maintain a high inhibitory efficacy, and elucidated the underlying microbial mechanisms through in vitro fermentation and metagenomics analysis.

**Results:**

The combined treatment decreased CH_4_ production by 98.21% (*P* < 0.01) without impairing DM degradation, and markedly shifted rumen fermentation towards propionate, lowering the acetate-to-propionate ratio (1.59 vs. 2.65; *P* < 0.01). Metagenomic profiling revealed substantial reductions in the abundance of *Methanobrevibacter* and *Ruminococcus*, along with increased levels of propionate-associated bacteria such as *Prevotella*, *Treponema*, *Eubacterium*, and *Selenomonas* (*P* < 0.01). Functionally, the combined treatment downregulated key enzymes in hydrogenotrophic and methylotrophic methanogenesis, including methyl-coenzyme M reductase (EC:2.8.4.1) and tetrahydromethanopterin S-methyltransferase (EC:2.1.1.86), thereby blocking terminal methanogenic steps.

**Conclusions:**

Collectively, these results demonstrate that co-supplementation with *A. taxiformis* and 3-NOP achieves near-complete methanogenesis inhibition at drastically reduced seaweed dosage through coordinated changes in fermentation patterns, microbial community structure, and methanogenic enzymatic pathways. This approach provides a practical strategy to overcome biomass limitations of *A. taxiformis* and warrants validation in long-term in vivo trials.

**Supplementary Information:**

The online version contains supplementary material available at 10.1186/s40104-026-01430-x.

## Introduction

The increasing concentrations of atmospheric greenhouse gases (GHG) are a primary driver of global warming. Among them, CH_4_ is considered the second most impactful GHG after carbon dioxide (CO_2_) [1]. Although global initiatives aim to lower CH_4_ emissions by approximately 30%, as proposed in international climate mitigation frameworks, ongoing mitigation efforts remain insufficient to meet the targets outlined in the Paris Agreement [2]. CH_4_ emissions from the rumens of ruminant livestock account for about 14.5% of global anthropogenic GHG emissions and 40% of all livestock emissions [3]. Therefore, developing effective strategies to reduce CH_4_ emissions from livestock, particularly from ruminant enteric fermentation, is crucial for global warming mitigation efforts.

Among various proposed interventions, the red seaweed *Asparagopsis taxiformis* has emerged as a highly promising feed additive to effectively reduce enteric CH_4_ emissions in ruminant livestock [4, 5]. Both in vitro and in vivo studies have revealed that adding ≤ 5% *A. taxiformis* to dry matter (DM) as a feed additive can suppress CH_4_ emissions by up to 99%, demonstrating a substantial CH_4_ emission reduction potential [6–9]. The anti-methanogenic mechanism of action of *A*. *taxiformis* is multifaceted. Primarily, its bioactive compounds (e.g., bromoform) act as analogs of methyl-coenzyme M, competitively inhibiting the enzyme methyl-coenzyme M reductase (MCR), which is essential for the final step of methanogenesis. Additionally, *A*. *taxiformis* supplementation alters metabolic pathways in the rumen, diverting hydrogen (H_2_) away from methanogenesis towards alternative sinks, such as propionate fermentation (propionogenesis) [6, 7]. This change in rumen metabolism can also lead to a direct reduction in the abundance of methanogenic archaea, particularly those from the genus *Methanobrevibacter* [10–12]. Our previous research has demonstrated that inclusion of 5% *A. taxiformis* reduced CH_4_ emissions by 98.53%, and identified the inhibition of MCR activity as the primary mechanism underlying its CH_4_ emission reduction potential [13]. However, the widespread application of *A*. *taxiformis* faces significant challenges. Its natural distribution is restricted to tropical and warm-temperate waters [14], and large-scale cultivation remains difficult due to its complex life cycle and specific environmental requirements. Consequently, the limited natural harvest and aquaculture production cannot meet the growing demand from the global ruminant livestock industry for effective CH_4_ emission reduction.

Another widely studied synthetic methane inhibitor is 3-nitrooxypropanol (3-NOP), which has already been applied in many countries around the world and also garnered significant interest due to its analogous function in targeting and inhibiting MCR [14]. Previous studies have shown that 3-NOP is a structural analog of methyl-coenzyme M and can bind preferentially to MCR, catalyzing nickel oxidation and inactivating MCR to inhibit CH_4_ production [15]. A recent meta-analysis confirmed its efficacy, reporting an average reduction of 32.7% in methane production [16]. Dietary supplementation with 3-NOP typically reduces enteric methane emissions by 26% to 54%, which is much lower than the reduction achieved with *A*. *taxiformis* [17, 18]. Liu et al. [19] reported that supplementing 3-NOP at a concentration of 2 mg/g during in vitro fermentation decreased CH_4_ emissions by 9.71%, and when combined with fumarate, the reduction increased to 11.48%, suggesting that a combined supplementation strategy may enhance methane mitigation efficacy. To maintain the high inhibitory efficiency of *A. taxiformis* on CH_4_ emissions while reducing its required dosage, a co-supplementation experiment was performed using both *A. taxiformis* and 3-NOP.

Supplementation level is a major factor influencing the potential for CH_4_ emission reduction of *A*. *taxiformis* and 3-NOP, and combined supplementation may be an effective means of resolving the dilemma of *A*. *taxiformis* biomass limitation and limited CH_4_ reduction magnitude by 3-NOP. Our preliminary experimental results indicated that reducing the supplementation levels of *A. taxiformis* and 3-NOP to 0.32% and 0.05%, respectively, still effectively lowered CH_4_ emissions, confirming the dominant role of *A. taxiformis* in methane emission reduction. However, a knowledge gap remains regarding the impact of this intervention on rumen fermentation, necessitating further investigation. Therefore, the objectives of this study were to evaluate the effects of combined supplementation with *A. taxiformis* and 3-NOP on CH_4_ emissions and ruminal fermentation parameters, and to elucidate the underlying microbial mechanisms involved in CH_4_ reduction through a metagenomic approach.

## Materials and methods

### Experimental preparation

The *A*. *taxiformis* used in this study was collected from the coastal waters of Naozhou Island (20°54'–21°10' N, 109°00'–109°15' E; Zhanjiang, Guangdong Province, China). Upon arrival at the laboratory, the seaweed was briefly rinsed in fresh water for 1 min to remove silt, salt, and other impurities. Subsequently, surface moisture was removed using a mechanical roller dryer. The rinsed biomass was then completely dehydrated by freeze-drying for 48 h. The resulting lyophilized material was ground using a mill and sieved through a 1-mm screen. The processed *A*. *taxiformis* powder was stored in airtight containers at −20 °C until further analysis. Our previous study identified several major bioactive compounds in *A*. *taxiformis*, including bromoacetic acid (10.28%), catechin (6.25%), rosmarinic acid (3.48%), dibromoiodomethane (2.91%), coumaric acid (2.71%), P-bromophenol (2.36%), hesperidine (2.32%), 2,4,6-tribromothiophenol (2.28%), daidzein (2.26%), 2,4-dibromophenol (1.78%) [13]. Bromomethane, which has been reported as a key anti-methanogenic compound in *A. taxiformis*, was not detected in the present analysis, possibly due to its high volatility and instability during sample handling and extraction procedures. The synthetic methane inhibitor 3-NOP, with a stated purity of > 98%, was procured from Shanghai Aladdin Biochemical Technology Co., Ltd. (Shanghai, China).

### Experimental design

An in vitro fermentation experiment was conducted to evaluate the effects of combined supplementation with *A. taxiformis* and 3-NOP. Two experimental treatments were evaluated: (1) a control (CON) with no additives, and (2) a combined treatment (AN) supplemented with both *A. taxiformis* (3.20 mg/g DM; 0.32%) and 3-NOP (0.50 mg/g DM; 0.05%). The supplementation levels of *A*. *taxiformis* and 3-NOP used in the present study were determined based on a preliminary orthogonal design experiment. The experimental design and main results of the orthogonal test are presented in Table S1. The fermentations were run for 48 h. Each treatment had 6 replicates, resulting in a total of 12 fermentation units.

### In vitro fermentation

Three Holstein cows were selected as rumen fluid donors in the morning of the experiment. The cows were 3-year-old lactating individuals with an average body weight of 550 ± 30 kg. All animals were maintained under similar management conditions and fed a total mixed ration formulated to meet their nutrient requirements, with a forage-to-concentrate ratio of approximately 40:60 (dry matter basis). The cows had free access to water.

Rumen fluid was collected 2 h after morning feeding, filtered through 4 layers of gauze, mixed in equal volumes, and placed in a preheated thermos bottle and brought back to the laboratory within 30 min. The rumen fluid was mixed with the buffer solution [20] (1:2, vol/vol) and stirred continuously in a 39 °C water bath to ensure uniform mixing. The process was run under carbon dioxide flushing to maintain an anaerobic environment. The fermentation substrate was the same as the dairy cow diet, dried at 65 °C for 48 h, and then milled to pass through a 1-mm screen. The 500 mg of fermentation substrate and 75 mL of mixed artificial rumen fluid were added to 100-mL fermentation bottles. The chemical compositions of the fermentation substrates and *A*. *taxiformis* are consistent with those of our previous research (Table S2). *A. taxiformis* and 3-NOP were supplemented in the treatment group, but not in the control group. The fermentation bottles were then sealed with butyl plugs and aluminum foil. This process was also carried out under CO_2_ flushing. To simulate the fermentation environment inside the rumen as closely as possible, all fermentation flasks were incubated for 48 h in a constant-temperature water bath shaker set at 39 °C and 85 r/min [21]. When the fermentation time reached 48 h, the fermentation bottle was placed in an ice water bath to stop the fermentation and collect samples for subsequent analysis.

### Experimental sample collection and analysis

At 2, 4, 8, 12, 24, and 48 h of the experiment, the gas was collected using a standard 30 mL syringe and stored in a 300-mL aluminum foil gas collection bag. Regularly release gas to prevent excessive accumulation of gas in the fermentation bottles and maintain conditions similar to normal rumen fermentation. This study focuses on the overall fermentation results after 48 h of cultivation, and does not concern the changes in fermentation over time. The gas components in the collected gas samples were measured using the SP-2060 T gas chromatograph (Tianpu, China). The remaining biomass in the fermentation bottle was collected using a nylon bag, and each nylon bag was rinsed with tap water until the water flow was clear, then dried at 65 °C for 48 h, and the dry matter degradation (DMD) was calculated. After the fermentation bottle was cooled for 30 min, liquid samples (2 × 5 mL) were collected and stored at −80 °C for subsequent volatile fatty acid (VFA) and metagenomic analysis. The liquid sample was centrifuged at 3,200 × *g* for 10 min, and 1 mL of the supernatant was collected and mixed with 0.2 mL of 25% metaphosphate, and then placed in an ice water bath for more than 30 min. Centrifuged again at 10,000 r/min for 10 min, and the supernatant was stored at −20 °C. The VFA concentration was measured using Agilent 6890 N (Agilent, USA).

### DNA extraction, library construction, and metagenomic sequencing

Total genomic DNA was extracted using the E.Z.N.A.^®^ Soil DNA Kit (Omega Bio-Tek, Norcross, GA, USA) following the manufacturer’s protocol. DNA concentration and purity were assessed using a Synergy HTX plate reader and NanoDrop 2000 spectrophotometer (Thermo Fisher Scientific, USA), respectively. Sequencing libraries were constructed from ~350 bp DNA fragments generated by the Covaris M220 ultrasonicator (Gene Company Limited, China) using the NEXTflex™ Rapid DNA-Seq Kit (Bioo Scientific, Austin, TX, USA), and paired-end sequenced on the Illumina NovaSeq 6000 platform (NovaSeq Reagent Kits; Illumina Inc., San Diego, CA, USA) at Majorbio Bio-Pharm Technology Co., Ltd. (Shanghai, China).

Raw reads were processed using fastp (https://github.com/OpenGene/fastp, version 0.23.0) [22] for adapter trimming and quality filtering (average quality score > 20, read length > 50 bp). Host sequences were removed by mapping to the *Bos taurus* reference genome (NCBI database, ARS-UCD 2.0) using BWA (http://bio-bwa.sourceforge.net, version 0.7.17) [23]. Assembly was performed using MEGAHIT (https://github.com/voutcn/megahit, version 1.1.2) [24], and open reading frames (ORFs) were predicted with Prodigal (https://github.com/hyattpd/Prodigal, version 2.6.3) [25]. A non-redundant gene catalog was generated using CD-HIT (http://weizhongli-lab.org/cd-hit/, version 4.6.1) at 90% sequence identity and 90% coverage [26]. Post-assembly, reads were mapped to the non-redundant gene catalog using SOAPaligner (https://github.com/ShujiaHuang/SOAPaligner, version 2.21) with a minimum identity threshold of 95% [27]. Taxonomic and functional annotation were conducted using DIAMOND (http://ab.inf.uni-tuebingen.de/software/diamond/, version 2.0.13) [28] against the NCBI NR (accessed August 30, 2023), and Kyoto Encyclopedia of Genes and Genomes (KEGG).

### Statistical analysis

Data on fermentation parameters, including dry matter degradation rate, total gas production (TGP), gas composition (CH_4_, CO_2_, H_2_), and volatile fatty acids, were statistically analyzed using SPSS 27.0 software. Measurement information was expressed as mean ± standard deviation. Independent samples *t*-test was used for comparison between groups. Normality of data was first assessed by Shapiro–Wilk test, and variance alignment was assessed by Levene test. *P* < 0.05 was considered statistically significant. Metagenomic sequencing and initial data processing were performed by Shanghai Meiji Biomedical Technology Co., Ltd. (Shanghai, China). Subsequent bioinformatic analysis, including taxonomic and functional annotation, was conducted on the Majorbio Cloud Platform (https://cloud.majorbio.com; accessed on 10 May 2024).

## Results

### Effects of combined supplementation with *A. taxiformis* and 3-NOP on gas production and fermentation parameters

#### Gas production parameters

**Fig. 1:**
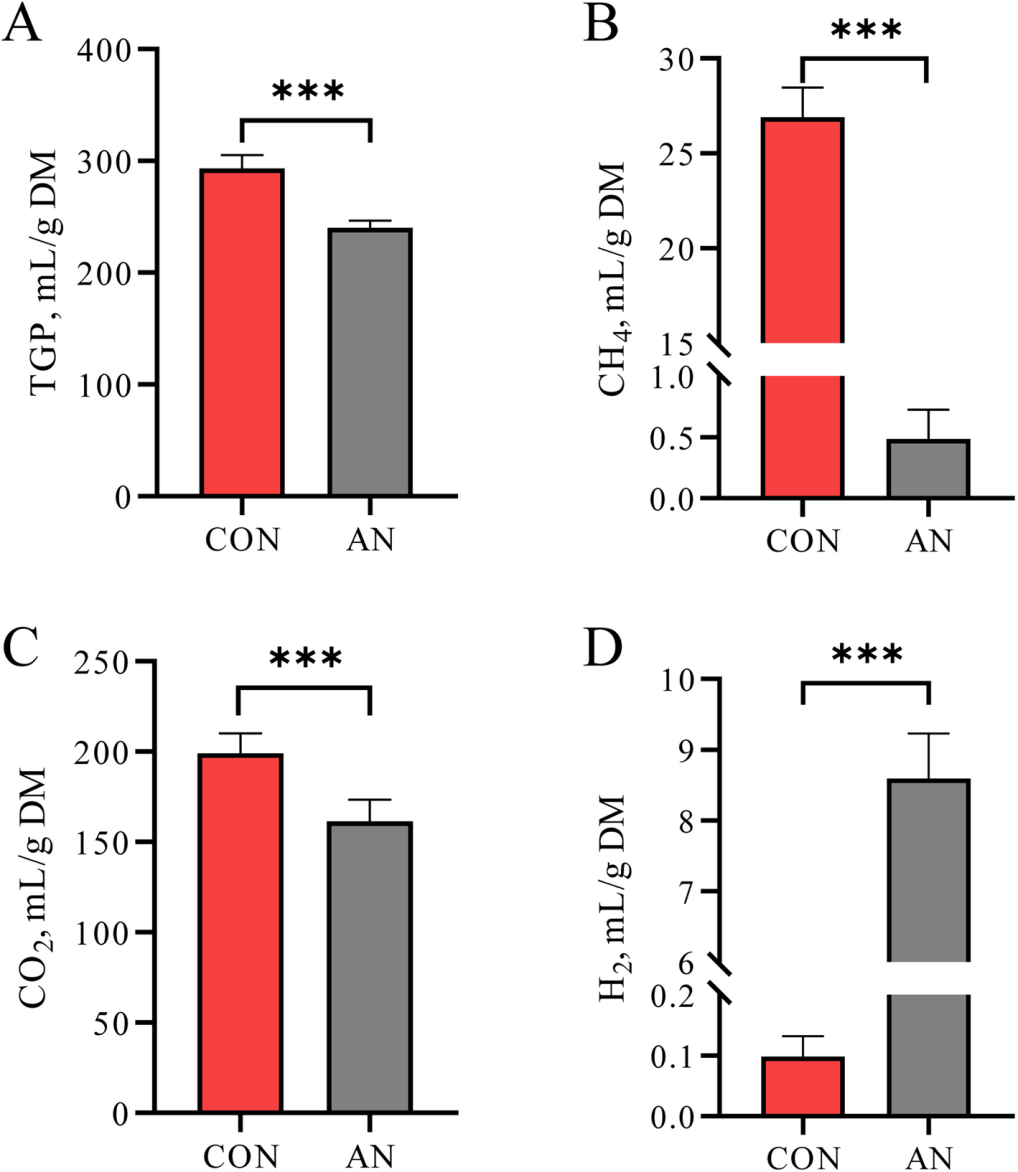
Effects of the combined supplementation on gas production parameters. **A** Total gas production. **B** CH_4_ production. **C** CO_2_ production. **D** H_2_ production. CON, control group; AN, CON plus 0.32% *A. taxiformis* and 0.05% 3-NOP; ^*^P < 0.05, ^**^P < 0.01, ^***^P < 0.001

All gas production parameters are expressed per gram of DM of fermented substrate. Total gas produced TGP was significantly lower in the AN group than in the CON group (240.02 ± 6.40 vs. 293.03 ± 12.02 mL/g DM, *P* < 0.01; Fig. 1A). CH_4_ production was substantially reduced in the AN group, with a near-complete inhibition to 0.48 ± 0.24 mL/g DM compared to 26.89 ± 1.56 mL/g DM in the CON group (*P* < 0.01; Fig. 1B). This represents a 98.21% reduction in CH_4_ emissions. Similarly, CO_2_ production was lower in the AN group than in the CON group (161.31 ± 12.05 vs. 199.02 ± 10.99 mL/g DM, *P* < 0.01; Fig. 1C). CH_4_ production was markedly reduced in the AN group, and was accompanied by significantly higher H_2_ production compared to the CON group (8.58 ± 0.64 vs. 0.09 ± 0.03 mL/g DM, *P* < 0.01; Fig. 1D).

#### Fermentation characteristics

The effects of combined *A*. *taxiformis* and 3-NOP supplementation on rumen fermentation characteristics are presented in Table 1. DMD was not affected by the combined treatment, as indicated by the determined DMD rates of 85.08% ± 2.31% and 85.87% ± 2.13% for the CON and AN group, respectively (*P* = 0.55). However, the total volatile fatty acid (TVFA) concentration was significantly lower in the AN group than in the CON group (57.73 ± 2.59 vs. 72.57 ± 7.82 mmol/L, *P* < 0.01). The concentrations of acetate, butyrate, and isobutyrate were lower in the AN group (30.60 ± 1.04, 5.08 ± 0.32, and 0.44 ± 0.03 mmol/L, respectively) than in the CON group (43.73 ± 5.04, 8.09 ± 0.83, and 1.08 ± 0.10 mmol/L, respectively) (*P* < 0.01). In contrast, the concentrations of propionate and valerate were higher in the AN group (19.16 ± 1.03 and 2.44 ± 0.19 mmol/L, respectively) than in the CON group (16.45 ± 1.53 and 1.35 ± 0.11 mmol/L, respectively) (*P* < 0.01). The significantly lower acetate concentration and higher propionate concentration resulted in a lower acetate-to-propionate ratio (A:P ratio) in the AN group (1.59 ± 0.03) compared to the CON group (2.65 ± 0.06) (*P* < 0.01).

**Table 1:** Effects of the combined supplementation of *A. taxiformis* and 3-NOP on the DMD and VFA profiles in vitro

Item	CON	AN	*P*
DMD, %	85.08 ± 2.31	85.87 ± 2.13	0.55
TVFA, mmol/L	72.57 ± 7.82^A^	57.73 ± 2.59^B^	< 0.01
Acetate, mmol/L	43.73 ± 5.04^A^	30.60 ± 1.04^B^	< 0.01
Propionate, mmol/L	16.45 ± 1.53^B^	19.16 ± 1.03^A^	< 0.01
Isobutyrate, mmol/L	1.08 ± 0.10^A^	0.44 ± 0.03^B^	< 0.01
Butyrate, mmol/L	8.09 ± 0.83^A^	5.08 ± 0.32^B^	< 0.01
Valerate, mmol/L	1.35 ± 0.11^B^	2.44 ± 0.19^A^	< 0.01
A:P ratio	2.65 ± 0.06^A^	1.59 ± 0.03^B^	< 0.01

*CON* Control group, *AN* CON plus 0.32% *A. taxiformis* and 0.05% 3-Nitrooxypropanol, *A:P ratio* Acetate/Propionate ratio

^A,B^Means bearing different superscripts in the same row differ significantly (*P* < 0.01)

### Effects of combined supplementation with *A. taxiformis* and 3-NOP on microbial community composition

#### Bacterial community diversity and composition

**Fig. 2:**
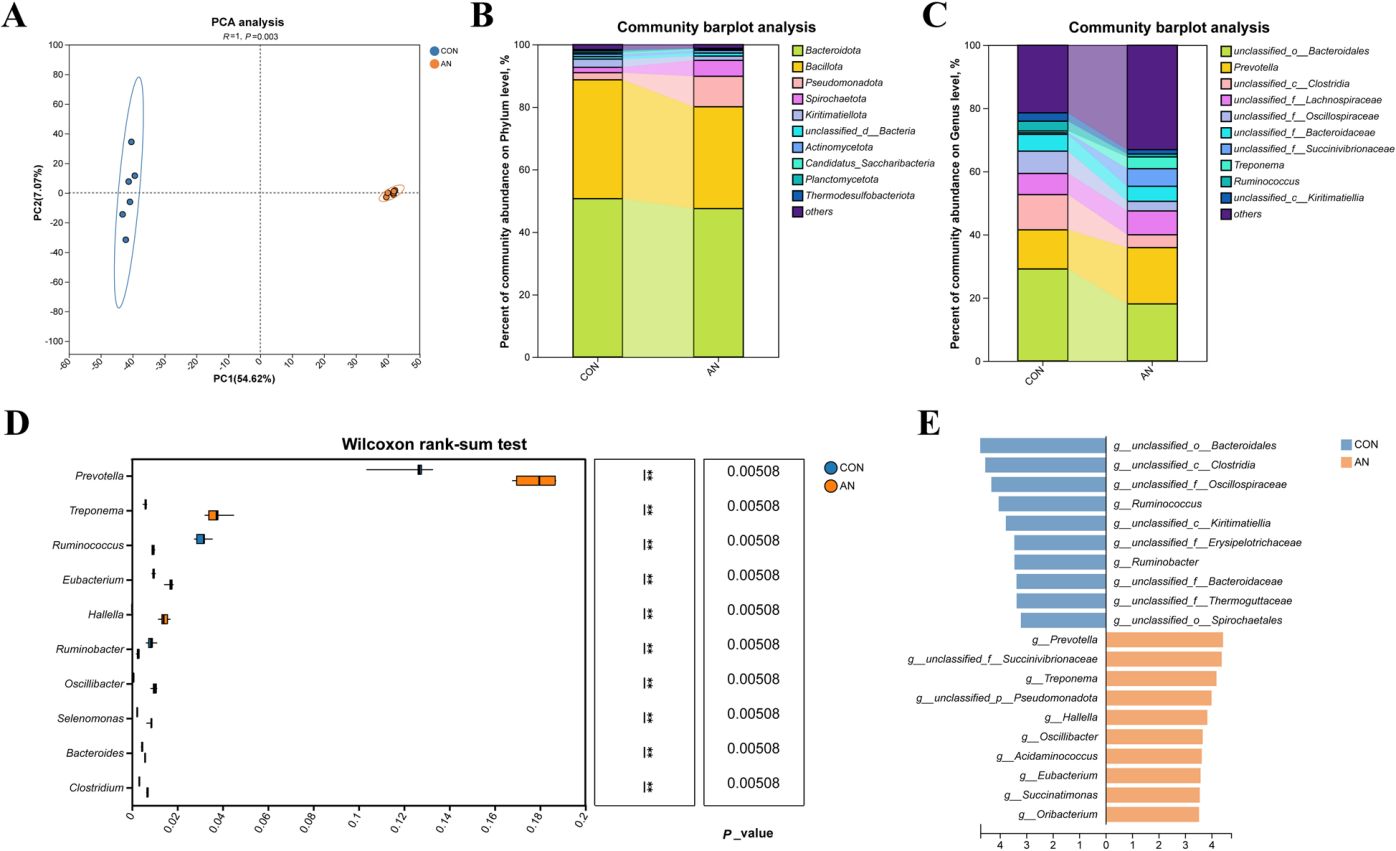
Effects of the combined supplementation on rumen bacterial composition during in vitro rumen fermentation. **A** Beta diversity. **B** Relative abundances of the 10 most abundant phyla. **C** Relative abundances of the 10 most abundant genera. **D** Differences in bacterial genus levels by metagenomics sequencing. **E** The LDA values of different species among the two groups on genus level (LDA > 2). CON, control group; AN, CON plus 0.32% *A. taxiformis* and 0.05% 3-NOP; ^*^P < 0.05, ^**^P < 0.01, ^***^P < 0.001

The α-diversity indices of the bacterial community are summarized in Table 2. Specifically, the richness indices ACE and Chao1 and the Simpson index were significantly lower in the AN group than in the CON group (*P* < 0.01), whereas the Shannon index was significantly higher in the AN group (*P* < 0.01). These results indicate that the combined supplementation significantly reduced bacterial community richness but increased its evenness. Principal component analysis (PCA), based on genus-level composition, revealed a clear separation between the CON and AN group (Fig. 2A). This separation was confirmed to be statistically significant by an analysis of similarity (ANOSIM) test (*P* < 0.01). Metagenomic analysis was used to evaluate the effects of the combined supplementation on microbial composition. At the phylum level, the most abundant phyla were Bacteroidota, Bacillota, and Pseudomonadota collectively accounting for the majority of the bacterial community (Fig. 2B). At the genus level, *Prevotella*, *Treponema*, and *Ruminococcus* were identified as the dominant genera across all samples (Fig. 2C).

**Table 2:** Alpha diversity indices of bacterial and archaeal communities on the combined supplementation of *A. taxiformis* and 3-NOP

Item	CON	AN	*P*
Bac			
ACE	2735.66 ± 38.84^A^	2387.66 ± 32.69^B^	< 0.01
Chao 1	2735.66 ± 38.85^A^	2387.66 ± 32.70^B^	< 0.01
Shannon	2.89 ± 0.01^B^	3.38 ± 0.03^A^	< 0.01
Simpson	0.13^A^	0.08^B^	< 0.01
Arc			
ACE	111.16 ± 2.63^A^	92.00 ± 4.24^B^	< 0.01
Chao 1	111.16 ± 2.64^A^	92.00 ± 4.25^B^	< 0.01
Shannon	0.34 ± 0.03^B^	1.20 ± 0.03^A^	< 0.01
Simpson	0.89 ± 0.01^A^	0.50 ± 0.01^B^	< 0.01

*CON* Control group, *AN* CON plus 0.32% *A. taxiformis* and 0.05% 3-Nitrooxypropanol, *Bac* Bacterial, *Arc* Archaea, *ACE* Abundance-based coverage estimator

^A,B^Means bearing different superscripts in the same row differ significantly (*P* < 0.01)

Differential abundance analysis at the genus level was performed using the Wilcoxon rank-sum test. The AN group showed a significant increase in the relative abundance of several genera, including *Prevotella*, *Treponema*, *Eubacterium*, *Hallella*, *Oscillibacter*, *Selenomonas*, *Bacteroides*, and *Clostridium* (*P* < 0.01; Fig. 2D), whereas the relative abundances of *Ruminococcus* and *Ruminobacter* were significantly decreased (*P* < 0.01; Fig. 2D). The linear discriminant analysis effect size (LEfSe) results (LDA > 2) further identified specific bacterial genera that were significantly enriched in the AN group, including *Prevotella*, *Treponema*, *Hallella*, and *Succinatimonas* (Fig. 2E).

#### Archaeal community diversity and composition

**Fig. 3:**
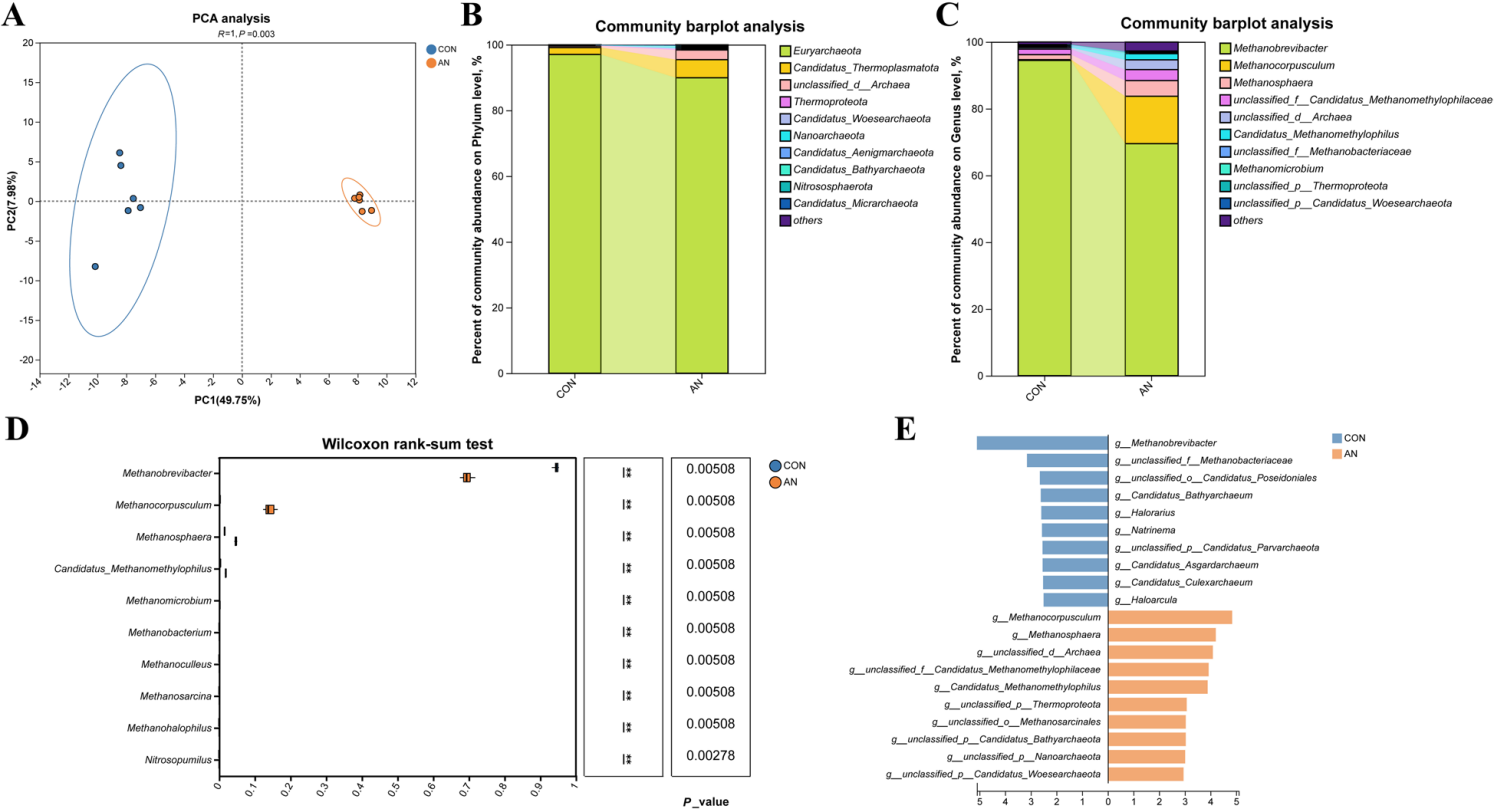
Effects of the combined supplementation on rumen archaeal composition during in vitro rumen fermentation. **A** Beta diversity. **B** Relative abundances of the 10 most abundant phyla. **C** Relative abundances of the 10 most abundant genera. **D** Differences in archaeal genus levels by metagenomics sequencing. **E** The LDA values of different species among the two groups on genus level (LDA > 2). CON, control group; AN, CON plus 0.32% *A. taxiformis* and 0.05% 3-NOP; ^*^P < 0.05, ^**^P < 0.01, ^***^P < 0.001

The α-diversity of the archaeal community showed a response to the combined supplementation that was similar to the trends observed in the bacterial community (Table 2). Specifically, the ACE, Chao1, and Simpson indices were significantly lower (*P* < 0.01), whereas the Shannon index was significantly higher in the AN group compared to the CON group (*P* < 0.01). This pattern indicates a concomitant reduction in richness and an increase in evenness within the archaeal community following treatment. Consistent with the bacterial community findings, the archaeal community composition also underwent significant restructuring. The PCA results of the archaeal community showed a significant difference between the AN group and the CON group, which were clearly segregated (*P* < 0.01; Fig. 3A).

At the phylum level, Euryarchaeota was overwhelmingly dominant (Fig. 3B). At the genus level, the community was primarily comprised of *Methanobrevibacter*, *Methanocorpusculum*, and *Methanosphaera* (Fig. 3C). The AN group significantly increased the relative abundance of *Methanocorpusculum*, *Methanosphaera*, and *Methanomethylophilus* (*P* < 0.01), whereas the relative abundance of *Methanobrevibacter* was decreased compared to the CON group (*P* < 0.01; Fig. 3D). Archaea such as *Methanocorpusculum* and *Methanosphaera* were significantly enriched in the AN group when LDA > 2 (Fig. 3E).

#### Functional profiling via KEGG pathway enrichment analysis

**Fig. 4:**
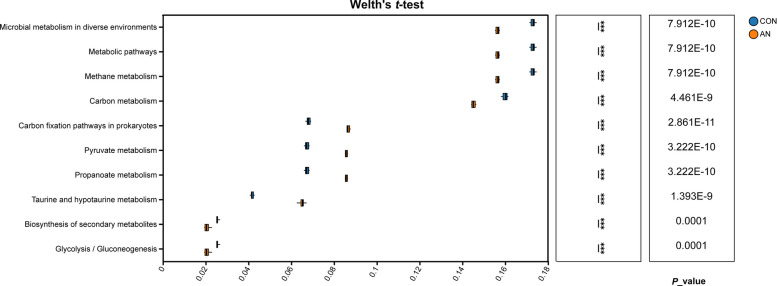
Effects of the combined supplementation on KEGG pathway during in vitro rumen fermentation. CON, control group; AN, CON plus 0.32% *A. taxiformis* and 0.05% 3-NOP; ^*^P < 0.05, ^**^P < 0.01, ^***^P < 0.001

To elucidate the functional mechanisms underlying the reduction of CH_4_ production, metagenomic data were annotated against the Kyoto Encyclopedia of Genes and Genomes (KEGG) database to compare microbial metabolic pathways between groups. Comparative analysis of level 3 KEGG pathways revealed significant differences between the CON and AN group (Fig. 4). Among the top 10 most enriched pathways, the relative enrichment of six pathways was significantly lower in the AN group: microbial metabolism in diverse environments, metabolic pathways, methane metabolism, carbon metabolism, biosynthesis of secondary metabolites, and glycolysis/gluconeogenesis (*P* < 0.01). Conversely, the AN group had a higher relative enrichment of carbon fixation pathways in prokaryotes, pyruvate metabolism, propanoate metabolism, and taurine and hypotaurine metabolism compared to the CON group (*P* < 0.01).

**Fig. 5:**
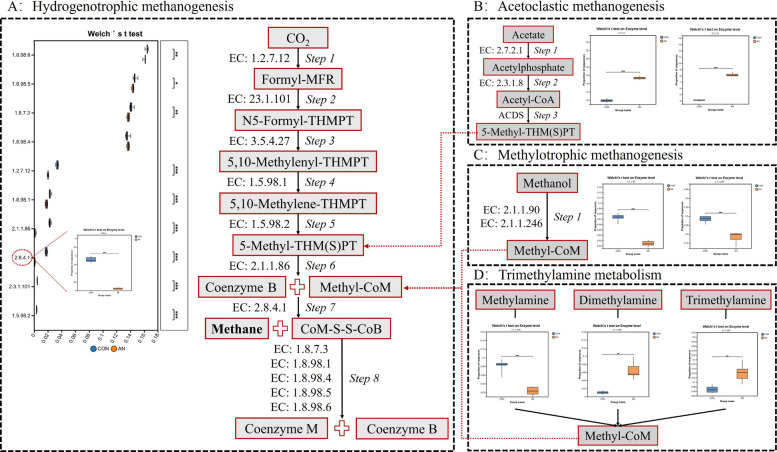
Comparison of gene abundance associated with enzymes involved in different methanogenesis pathways across two treatments. **A** Hydrogenotrophic methanogenesis. **B** Acetoclastic methanogenesis. **C** Methylotrophic methanogenesis. **D** Trimethylamine metabolism. CON, control group; AN, CON plus 0.32% *A. taxiformis* and 0.05% 3-NOP; ^*^P < 0.05, ^**^P < 0.01, ^***^P < 0.001

To identify the specific enzymatic targets within the methane metabolism pathway, we quantified the relative abundance of genes encoding key enzymes. In the AN group, the relative abundance of genes encoding multiple key enzymes involved in the hydrogenotrophic methanogenesis pathway was significantly reduced, including: EC:1.8.98.6, EC:1.8.7.3, EC:1.2.7.12, EC:1.8.98.1, EC:2.1.1.86, EC:2.8.4.1, EC:2.3.1.101, EC:1.5.98.2 (*P* < 0.01), and EC:1.8.98.5 (*P* < 0.05) (Fig. 5A). In contrast, the abundance of genes encoding enzymes (EC:2.7.2.1 and EC:2.3.1.8) involved in the acetoclastic methanogenesis pathway was significantly increased (Fig. 5B;* P* < 0.01). Moreover, the abundance of genes encoding enzymes (EC:2.1.1.90, EC:2.1.1.246, and EC:2.1.1.248) involved in the methylotrophic methanogenesis pathway (utilizing methanol and methylamines) was significantly decreased (*P* < 0.01), while the abundance of genes encoding enzymes (EC:2.1.1.249 and EC:2.1.1.250) involved in methylotrophic methanogenesis was significantly increased (*P* < 0.01; Fig. 5C and D).

#### Exploring potential connection mechanisms by correlation network analysis

**Fig. 6:**
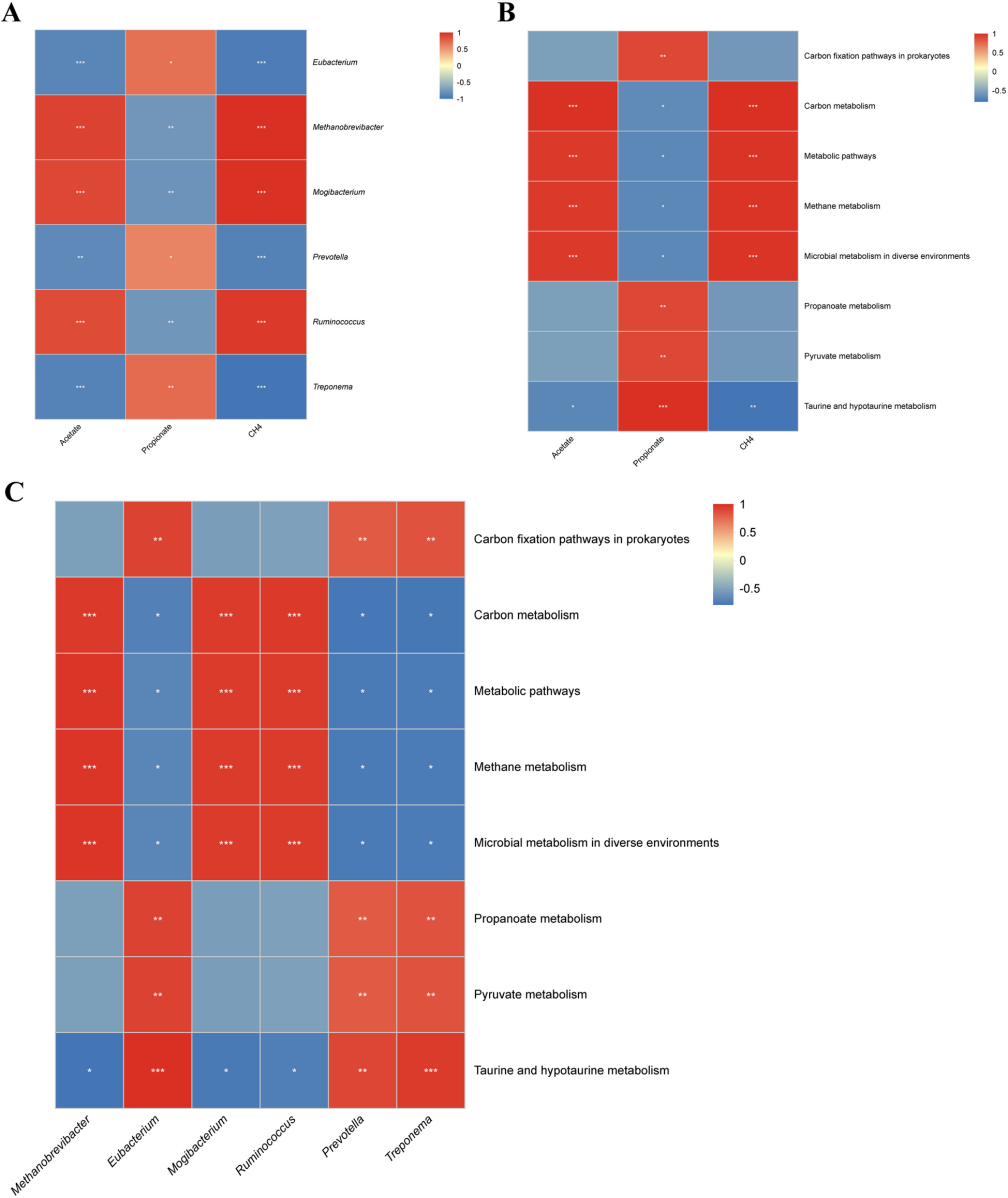
Correlation analysis shows the relationships between the rumen fermentation parameters, microorganisms and functional pathway. **A** The correlation analysis between rumen fermentation parameters and microorganisms. **B** The correlation analysis between rumen fermentation parameters and functional pathway. **C** The correlation analysis between microorganisms and functional pathway. CON, control group; AN, CON plus 0.32% *A. taxiformis* and 0.05% 3-NOP; ^*^P < 0.05, ^**^P < 0.01, ^***^P < 0.001

To integrate the observed changes and elucidate potential mechanisms, a correlation network analysis was performed between key rumen fermentation parameters, microbial species, and KEGG pathways previously identified to be significantly altered. As shown in Fig. 6A, a nearly identical, correlation pattern was observed between methane yield and these microbial species. Acetate concentration had significant positive correlations with the abundances of *Methanobrevibacter*, *Mogibacterium*, and *Ruminococcus* (*P* < 0.01), whereas it showed significant negative correlations with *Eubacterium*, *Prevotella*, and *Treponema* (*P* < 0.01). In stark contrast, propionate concentration showed a strong inverse correlation pattern: it was negatively correlated with *Methanobrevibacter*, *Mogibacterium*, and *Ruminococcus* (*P* < 0.01), and positively correlated with *Eubacterium* (*P* < 0.05), *Prevotella* (*P* < 0.05), and *Treponema* (*P* < 0.01).

The results of the correlation analysis of methanogenesis related fermentation parameters with the KEGG pathway are shown in Fig. 6B. The results reveal a consistent trend of correlation between CH_4_ and acetate, both positively correlated with methane metabolism, carbon metabolism, metabolic pathways, and microbial metabolism in diverse environments (*P* < 0.01), but negatively correlated with taurine and hypotaurine metabolism (*P* < 0.01). In contrast, propionate concentration was positively correlated with carbon fixation pathways in prokaryotes, pyruvate metabolism, propanoate metabolism, and taurine and hypotaurine metabolism (*P* < 0.01), but negatively correlated with methane metabolism, carbon metabolism, metabolic pathways, and microbial metabolism in diverse environments (*P* < 0.05). Additionally, correlation analysis was extended to include the top six most abundant microbial species and the top eight most abundant KEGG pathways (Fig. 6C). The abundances of *Methanobrevibacter*, *Mogibacterium*, and *Ruminococcus* were positively correlated with methane metabolism, carbon metabolism, metabolic pathways, and microbial metabolism in diverse environments (*P* < 0.01), but negatively correlated with taurine and hypotaurine metabolism (*P* < 0.05). The remaining three genera (*Eubacterium*, *Prevotella*, and *Treponema*) showed a consistent but opposite correlation pattern. Their abundances were positively correlated with carbon fixation pathways in prokaryotes, pyruvate metabolism, propanoate metabolism, and taurine and hypotaurine metabolism (*P* < 0.01), and negatively correlated with methane metabolism, carbon metabolism, metabolic pathways, and microbial metabolism in diverse environments (*P* < 0.05).

**Fig. 7:**
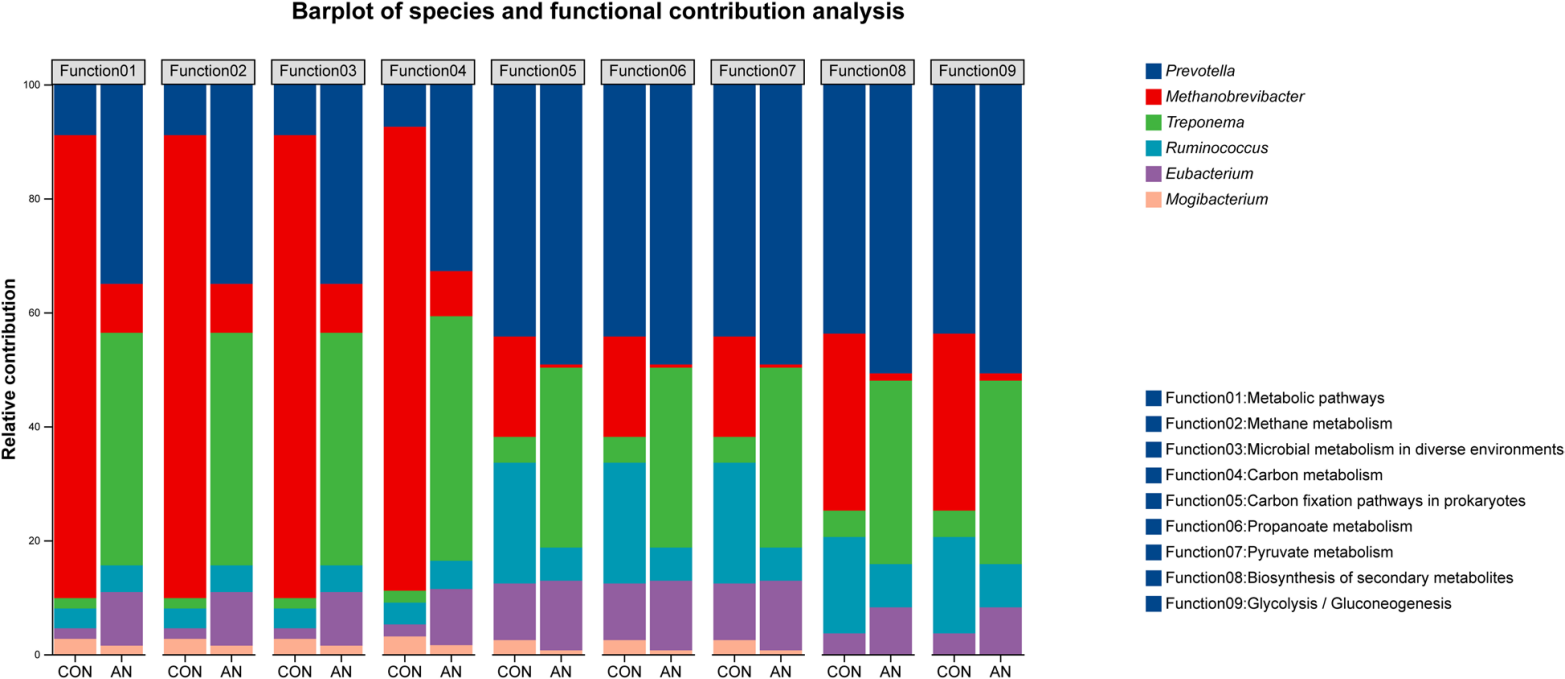
The distribution of the contribution of methanogenic species to the methanogenic function pathways. CON, control group; AN, CON plus 0.32% *A. taxiformis* and 0.05% 3-NOP

The relative contributions of these six genera to these nine metabolic functions are further visualized in Fig. 7. Results from this analysis indicated that the functional contributions of *Methanobrevibacter*, *Ruminococcus*, and *Mogibacterium* were lower in the AN group than in the CON group. Conversely, the functional contributions of *Prevotella*, *Treponema*, and *Eubacterium* were higher in the AN group. This finding suggests that the reduction in *Methanobrevibacter* abundance was the primary driver of CH_4_ reduction, with increased abundances of *Prevotella* and *Treponema* also playing significant roles. Additionally, the contributions of these microbial genera to individual KEGG enzymes are detailed in Table S3.

## Discussion

Previous studies have demonstrated that both *A*. *taxiformis* and 3-NOP are effective inhibitors of CH_4_ production when used individually in ruminant systems. However, information on their combined effects on rumen microbial community and methanogenesis is limited. In this study, combined supplementation with *A*. *taxiformis* and 3-NOP reduced CH_4_ emissions by 98.21%. In our previous study, supplementation with 5.00% (of DM) *A*. *taxiformis* reduced CH_4_ emissions by 98.53%, but the paradox of *A*. *taxiformis* restricted biomass and supplementation levels precluded its widespread application [13]. A major limitation for the practical application of *A. taxiformis* is the relatively high supplementation levels that are often required, while the harvestable algal biomass of this seaweed remains limited. Notably, this study achieved a comparable anti-methanogenic effect while substantially reducing the inclusion level of *A*. *taxiformis* (0.32% vs. 5.00%) through combined supplementation with 3-NOP.

The contrasting responses of DMD and VFA production indicate a change in the ruminal fermentation pattern in the AN group, suggesting that while the overall substrate degradation was maintained, the metabolic pathways responsible for the formation of fermentation end products were altered. The reduced production of VFAs, particularly acetate and butyrate, indicates that fermentation pathways were altered. Since acetate and butyrate production are major H_2_ generating pathways, their reduced production likely decreased H_2_ formation, thereby limiting substrate availability for methanogenesis [29]. This mechanism may partly explain the observed reduction in CH_4_ emissions. Conversely, only a modest increase in propionate production was observed, and the increase in valerate was relatively small and likely contributed little to the overall fermentation energy balance. Consistently, the decrease in the A:P ratio was mainly driven by a reduction in acetate concentration rather than a substantial increase in propionate. Propionate formation is an important pathway for H_2_ utilization and competes with CH_4_ production for available H_2_ [30, 31]. Similar responses have been reported in studies involving *A*. *taxiformis* [6] and 3-NOP [32] supplementation, but the relative contributions of the various pathways appear to vary across different studies. However, the modest increase in propionate observed in this study suggests that redirection of H_2_ to this pathway was limited and thus cannot fully explain the substantial decrease in CH_4_ production. From the perspective of energy metabolism, since VFAs are the primary metabolizable energy source for ruminants [33], the slight increases in propionate and valerate levels were insufficient to compensate for the lower energy utilization resulting from the decrease in acetate concentration, even though DMD remained unchanged. Conversely, reducing CH_4_ production helps improve overall energy conservation [34]. However, it remains unclear whether the reduction in CH_4_ emissions can fully compensate for the drop in VFA production. Elucidating the biological significance of these changes in the context of animal production performance and efficiency will require further research on combining fermentation properties with energy metabolism measurements. Despite the change in fermentation pathways, H_2_ accumulation remained significantly higher in the AN group. However, the ultimate fate of this accumulated H_2_ remains unclear. In vitro rumen fermentation allows H_2_ to accumulate in the gas phase more readily than under in vivo rumen conditions, where H_2_ is largely retained in the liquid phase and incorporated into reduced metabolic products [35]. Therefore, the accumulation of H_2_ observed in this study may partly reflect the intrinsic limitations of the in vitro system rather than the true H_2_ balance occurring in the rumen.

Rumen microorganisms play a major role in H_2_ utilization, and the changes in their community structure may contribute to this phenomenon [13]. The enrichment of bacterial genera such as *Prevotella*, *Eubacterium* and *Treponema* suggests that combined supplementation may promote alternative hydrogen-consuming pathways within the rumen microbial ecosystem. The negative correlation between these genera and CH_4_ production further supports the hypothesis that they may contribute to alternative H_2_ consumption pathways. This potential role is consistent with their positive correlations with the propanoate metabolism pathway. The ability of *Prevotella*, a dominant genus, to reduce CH_4_ production by degrading carbohydrates to produce propionate has been reported in studies on both *A*. *taxiformis* [36] and 3-NOP [37, 38]. Similarly, *Eubacterium* may compete with methanogens for H_2_ to produce fermentation end products, thereby possibly exerting an anti-methanogenic effect [39]. A similar negative correlation between *Eubacterium* abundance and CH_4_ production has also been reported in dairy cows supplemented with *A*. *taxiformis* [40], which is consistent with the patterns observed in the current study. Accordingly, these bacteria may be involved in alternative H_2_ consumption pathways, including propionate-related metabolic pathways, thereby reducing ruminal CH_4_ emissions.

Furthermore, we propose a mechanism of microbial interaction that may lead to changes in fermentation patterns. The enrichment of *Treponema* and *Clostridium* suggests a potential metabolic partnership: *Treponema* degrades carbohydrates to pyruvate via the Embden-Meyerhof-Parnas pathway [41], and the resulting pyruvate can subsequently be converted to propionate by *Clostridium* via the acrylate pathway [42]. However, this suggestion is based on indirect evidence and does not necessarily indicate the presence of a direct metabolic link with the production of propionate. This suggested interaction could contribute to redistributing carbon and hydrogen away from methanogenesis. This model is consistent with correlation analyses showing that *Treponema* abundance was negatively correlated with CH_4_ production, and positively correlated with both pyruvate and propanoate metabolism. Additionally, *Selenomonas* may be involved in other fermentation pathways, including succinate metabolism [43]. On the other hand, the decreased abundance of *Ruminococcus*, a key fiber-degrading bacterial genus that produces acetate and H₂, provides a complementary mechanism for the reduction of CH_4_ production [44]. Despite the reduction in classical fibrolytic bacteria such as *Ruminococcus*, DMD remained unchanged, suggesting that fiber degradation ability was maintained. This may be attributed to functional redundancy within the rumen microbiome, where other genera such as *Prevotella* and *Treponema*, which have hemicellulolytic and saccharolytic activities, could partially compensate for the loss of primary cellulolytic populations [45, 46].

The suppression of methanogenic archaea, particularly *Methanobrevibacter*, represents the most direct mechanism for the reduction in CH_4_ production observed in this study [47–49]. While the bacterial community changes may alter the H_2_ sink, the reduction in *Methanobrevibacter* abundance was the main cause of the significant decrease in CH_4_ production. The reduction of this major source of H_2_ consumption might explain the decrease in CH_4_ production and the occurrence of H_2_ accumulation during in vitro rumen fermentation. The strong positive correlation between *Methanobrevibacter* abundance and CH_4_ generation has been previously reported [50–52], and is consistent with the findings this study. Notably, the relative enrichment of the genera *Methanosphaera* and *Methanocorpusculum* may reflect a restructuring of the archaeal community under strong methanogenic inhibition. The former is the second largest contributor of CH_4_ after *Methanobrevibacter* in the rumen, but differs in that *Methanosphaera* consumes 4 mol of methanol to produce 3 mol of CH_4_ via the Methylotrophic pathway, and therefore, the higher its abundance, the lower the final emission of CH_4_, in the presence of a constant number of carbon sources [49, 53]. The *Methanocorpusculum* archaea are similarly recognized as “low CH_4_ emitters”, but their mechanism of action remains unclear [54]. Archaea are direct producers of CH_4_, and a decrease in *Methanobrevibacter* abundance and an increase in both *Methanosphaera* and *Methanocorpusculum* abundance directly reduce CH_4_ emissions.

To further understand the mechanisms underlying the inhibition of methanogenesis, genes involved in methane metabolism (ko00680) were examined, including those associated with hydrogenotrophic, acetoclastic, methylotrophic, and trimethylamine-dependent pathways. Among them, hydrogenotrophic methanogenesis is the dominant pathway in the rumen and is primarily carried out by *Methanobrevibacter*, which produces CH_4_ by reducing CO_2_ with H_2_. The observed decrease in *Methanobrevibacter* abundance thus suggests a direct disruption of the hydrogenotrophic methanogenesis pathway [40]. Consistently, many key enzymes involved in the early steps of this pathway, including formylmethanofuran dehydrogenase (EC:1.2.7.12), showed decreased abundance, indicating that the initial stages of the reduction of CO_2_ production were inhibited [55]. This inhibition likely limited the consumption of reducing equivalents, contributing to the reduction in CH_4_ production [56]. Additionally, the decreased abundance of enzymes involved in downstream hydrogenotrophic reactions suggests that the overall efficiency of CH_4_ formation was suppressed. Although acetoclastic methanogenesis can theoretically contribute to CH_4_ formation, it generally plays only a minor role in the rumen, and no dominant acetoclastic methanogens were found in the current dataset. Therefore, the substantial reduction in CH_4_ production observed in this study is more likely due to the inhibition of hydrogenotrophic methanogenesis.

Previous studies have highlighted the key role of MCR in the final step of CH_4_ production [56–58]. Halogenated metabolites produced by *A. taxiformis*, including bromoacetic acid and bromophenol derivatives, have been found to have antimicrobial properties and are thought to interfere with methanogenic activity by targeting this major enzymatic step [8, 59]. They are also thought to interfere with the MCR activity, the key enzyme catalyzing the final step of methanogenesis [6, 60]. The results of this study further support this proposed mechanism, as the combined supplementation was associated with reduced abundance of various enzymes involved in the final stages of methane metabolism, including tetrahydromethanopterin methyltransferase (EC:2.1.1.86) and MCR [61]. Such changes suggest that the methanogenesis pathway was suppressed not only at the methanogen abundance level, but also at the enzymatic level. Since MCR catalyzes the final reaction leading to CH_4_ formation, the inhibition of this step is likely a critical bottleneck in CH_4_ production [40]. Together with the upstream inhibition of hydrogenotrophic methanogenesis, this enzymatic inhibition may explain the near-total elimination of CH_4_ production observed in the combined supplementation treatment group. This multi-level inhibition of methanogenesis may partly explain the mechanism by which the combination of *A. taxiformis* and 3-NOP achieved a reduction in CH_4_ production comparable to that of high-dose *A. taxiformis* while using substantially lower supplementation levels, underscoring the potential of this combined treatment as a more effective CH_4_ production reduction approach. Although this *A. taxiformis* and 3-NOP supplementation has shown a considerable inhibitory effect on CH_4_ production in the in vitro rumen fermentation in this study, the long-term effect of this reaction on the rumen microbial community remains uncertain. The initial decrease in CH_4_ production might reflect the rapid response of microorganisms to these anti-methanogenesis compounds. However, the rumen microbial ecosystem has a high resilience and is capable of adapting to dietary or chemical disturbances [62]. Therefore, caution is needed when extrapolating the strong inhibitory effect observed in the present in vitro study to long-term in vivo conditions. Future studies combining long-term animal experiments with multi-omics approaches will be critical, as they will help assess the stability of microbial responses and their impact on the production performance of host animals.

This study primarily focused on bacterial and archaeal communities, but other important rumen microorganisms, such as protozoa and anaerobic fungi, were not evaluated. These microbial groups play major roles in fiber degradation, H_2_ production, and metabolic interactions with methanogens. Therefore, their omission limits a comprehensive ecosystem-level interpretation of the rumen microbial responses observed in this study. Further studies integrating various microbial domains will be necessary to fully elucidate the complex microbial interactions underlying the reduction of CH_4_ production.

## Conclusion

This study demonstrated that the combined supplementation with *A*. *taxiformis* (0.32% DM) and 3-NOP (0.05% DM) achieved near-complete reduction (98.21%) of CH_4_ emissions in vitro. The inhibitory effect observed with the combined supplementation appeared to involve various mechanisms, including the following: 1) restructuring the ruminal bacterial community to favor a pro-propionate fermentation pattern (e.g., increased abundance of *Prevotella* and decreased abundance of *Ruminococcus*), thereby lowering the A:P ratio and diverting H_2_ away from methanogenesis; and 2) directly inhibiting methanogenesis by reducing the abundance of *Methanobrevibacter* and levels of key enzymes (e.g., EC:2.1.1.86, EC:2.8.4.1, and EC:2.1.1.246) involved in hydrogenotrophic and methylotrophic pathways, ultimately limiting the MCR activity and obstructing the final step of CH_4_ production. These findings confirm that 3-NOP can synergize with *A*. *taxiformis* to drastically reduce the required effective dosage of the *A*. *taxiformis* while maintaining maximal efficacy. This strategy represents a highly promising approach to making the reduction of CH_4_ production in ruminants more feasible and economically viable. Further verification in long-term in vivo studies is necessary to confirm these benefits of this approach and assess its impacts on animal health, productivity, and product safety.

## Supplementary Information

Additional file 1: Table S1. Orthogonal experimental design and CH_4_ mitigation effects of different inclusion levels of *A. taxiformis* and 3-NOP in vitro.

Additional file 2: Table S2. Chemical composition of substrates and *A. taxiformis* used in the in vitro rumen fermentation.

Additional file 3: Table S3. Contributions of microbial genera to individual KEGG enzymes.

## Data Availability

Sequence data associated with this project have been deposited in the NCBI Short Read Archive database (PRJNA1363720). Other data generated or analyzed during this project are available from the corresponding author upon reasonable request.

## Abbreviations

3-NOP: 3-Nitrooxypropanol
AT: *Asparagopsis taxiformis*
DM: Dry matter
DMD: Dry matter degradation
GHG: Greenhouse gas
KEGG: Kyoto Encyclopedia of Genes and Genomes
MCR: Methyl-coenzyme M reductase
PCA: Principal component analysis
TGP: Total gas production
VFA: Volatile fatty acid
