# Supplementary material for: Near-complete inhibition of rumen methanogenesis via microbial and enzymatic modulation using a low dose of Asparagopsis taxiformis combined with 3-nitrooxypropanol
Source: J Anim Sci Biotechnol. 2026 Jun 5;17:110. doi: 10.1186/s40104-026-01430-x (PMC13237891; doi:10.1186/s40104-026-01430-x)
Supplement: Supplementary file 1 — Additional file 1: Table S1. Orthogonal experimental design and CH4 mitigation effects of different inclusion levels of A. taxiformis and 3-NOP in vitro. [file 40104_2026_1430_MOESM1_ESM.docx]

| Table S1. Orthogonal experimental design and CH_4_ mitigation effects of different inclusion levels of *A. taxiformis* and 3-NOP *in vitro*. | | | | | |
| --- | --- | --- | --- | --- | --- |
| Treat-ment | *A. taxiformis* (mg/g DM) | 3-NOP  (mg/g DM) | Total gas (mL/g DM) | CH₄ production (mL/g DM) | CH₄ reduction(%) |
| T0 | 0 | 0 | 273.84 | 26.42 | 0 |
| T1 | 0.0032 | 0.0005 | 237.58 | 0.79 | 97.02 |
| T2 | 0.0032 | 0.0010 | 236.95 | 0.39 | 98.53 |
| T3 | 0.0032 | 0.0015 | 255.58 | 0.50 | 98.11 |
| T4 | 0.0032 | 0.0020 | 247.71 | 0.61 | 97.70 |
| T5 | 0.0080 | 0.0010 | 242.59 | 0.31 | 98.81 |
| T6 | 0.0080 | 0.0005 | 236.89 | 0.54 | 97.94 |
| T7 | 0.0080 | 0.0020 | 233.94 | 0.30 | 98.88 |
| T8 | 0.0080 | 0.0015 | 241.59 | 0.31 | 98.83 |
| T9 | 0.0200 | 0.0015 | 239.49 | 0.25 | 99.06 |
| T10 | 0.0200 | 0.0020 | 232.76 | 0.32 | 98.79 |
| T11 | 0.0200 | 0.0005 | 228.39 | 0.26 | 99.02 |
| T12 | 0.0200 | 0.0010 | 236.51 | 0.37 | 98.61 |
| T13 | 0.0500 | 0.0020 | 231.44 | 0.17 | 99.35 |
| T14 | 0.0500 | 0.0015 | 225.28 | 0.17 | 99.37 |
| T15 | 0.0500 | 0.0010 | 226.33 | 0.28 | 98.94 |
| T16 | 0.0500 | 0.0005 | 230.95 | 0.25 | 99.04 |
