# Supplementary material for: Near-complete inhibition of rumen methanogenesis via microbial and enzymatic modulation using a low dose of Asparagopsis taxiformis combined with 3-nitrooxypropanol
Source: J Anim Sci Biotechnol. 2026 Jun 5;17:110. doi: 10.1186/s40104-026-01430-x (PMC13237891; doi:10.1186/s40104-026-01430-x)
Supplement: Supplementary file 2 — Additional file 2: Table S2. Chemical composition of substrates and A. taxiformis used in the in vitro rumen fermentation. [file 40104_2026_1430_MOESM2_ESM.docx]

Table S2. Chemical composition of substrates and *A. taxiformis* used in the *in vitro* rumen fermentation (DM basis).

| Item | Corn straw^1^ | Concentrate^2^ | *A. taxiformis* |
| --- | --- | --- | --- |
| DM % | 26.26 | 93.53 | NA |
| OM % | 92.85 | 92.81 | 63.17 |
| CP % | 7.12 | 20.47 | 22.61 |
| NDF % | 38.49 | 16.61 | 38.39 |
| ADF % | 21.69 | 6.22 | 13.23 |
| Ash % | 7.14 | 7.18 | 36.83 |

*A. taxiformis*, *Asparagopsis taxiformis*; DM, dry matter; OM, organic matter; CP, crude protein; NDF, neutral detergent fiber; ADF, acid detergent fiber; NA, not available. ^1^Corn stover harvested in experimental fields. ^2^Concentrate of composition: corn 500 g/kg, DDGS (Distiller’s dried grains with soluble) 235 g/kg, soybean meal 220 g/kg, stone powder 10 g/kg, dicalcium phosphate 9 g/kg, multivitamin 4 g/kg, salt 10 g/kg, multi-mineral 1 g/kg, baking soda 10 g/kg, mold inhibitor 1.5 g/kg.
